# Draft Genome Sequences of 10 Environmental *Pseudomonas aeruginosa* Strains Isolated from Soils, Sediments, and Waters

**DOI:** 10.1128/genomeA.00804-17

**Published:** 2017-08-24

**Authors:** Shailab D. Shrestha, David S. Guttman, Gabriel G. Perron

**Affiliations:** a Reem-Kayden Center for Science and Computation, Department of Biology, Bard College, Annandale-on-Hudson, New York, USA; b Department of Cell and Systems Biology, University of Toronto, Toronto, Ontario, Canada; c Centre for the Analysis of Genome Evolution and Function, University of Toronto, Toronto, Ontario, Canada

## Abstract

*Pseudomonas aeruginosa* is an important opportunistic pathogen that has the ability to grow in a range of environmental niches. Here, we report the draft genome sequences of 10 environmental strains of the bacterium isolated from soils, sediments, and waters in various locations in North America and South Africa.

## GENOME ANNOUNCEMENT

*Pseudomonas aeruginosa* is an important opportunistic pathogen often associated with multiple-drug resistance in hospital environments (1). The bacterium is especially problematic in immunocompromised individuals, such as patients with cystic fibrosis, in whom it causes severe health problems (2). In addition to human hosts, the bacterium has the ability to grow in a range of ecological niches, including plant and animal tissues of all kinds, as well as soils and aquatic environments (3). Even though *P. aeruginosa* demonstrates extensive genetic and phenotypic diversity in ecological niches (4, 5), the majority of whole-genome sequencing (WGS) studies considering the organism focus on medically important strains (6–9).

To investigate the genomic diversity of *P. aeruginosa* in the environment, we obtained the whole-genome sequences of 10 *P. aeruginosa* strains isolated from different locations across North America and South Africa. Strains Pae85, Pae100, Pae102, and Pae110 were isolated from soils and sediments at various sites around the greater Toronto area in Ontario, Canada, while Pae111, Pae112, and Pae113 were isolated from farm soil samples taken outside Maysville, KY. Strain Pae160 was isolated from a freshwater pond on a golf course in South Africa. Strains PaEB1 and PaEB6 were isolated from water sediment collected at two sites along the Saw Kill, a tributary of the Hudson River in Dutchess County, NY. The two strains were extracted from positive Pseudalert test kits (Idexx Laboratories, Westbrook, MA).

Isolated bacterial cultures were grown in Luria-Bertani broth overnight at 37°C in a shaking incubator. Cells were harvested and genomic DNA was extracted using the DNeasy UltraClean microbial kit (Qiagen, Carlsbad, CA). For the Pae strains, WGS was performed on a MiSeq platform at the University of Toronto Centre for the Analysis of Genome Evolution and Function (Toronto, Canada), according to the manufacturer’s protocol, to generate 250-bp paired-end reads. For the PaEB strains, WGS was performed on a HiSeq platform by Wright Labs at Juniata College (Huntingdon, PA), according to the manufacturer’s protocol, to generate 150-bp paired-end reads. For every strain, adapters were trimmed using Cutadapt version 1.14 (10) and quality filtered using Trimmomatic version 0.36 (11). Draft assemblies were conducted using SPAdes version 3.10.0 (12), with k-mer sizes of 21, 33, 55, 77, 99, and 127. Contigs shorter than 500 bp or with fewer than four reads were removed from the assembly. Finally, assembly improvement was undertaken as described by Page et al. (13), where contigs were scaffolded using SSPACE (14) and sequence gaps were filled using GapFiller (15). The assembly with the largest *N*_50_ value was used for further analysis.

The average number of contigs per genome was 80, with a standard deviation of 13.9. The draft genomes ranged in size from 6,336,070 bp to 6,836,824 bp, with an average G+C content of 66.3% (Table 1). The *N*_50_ of the draft genomes ranged from 121,945 bp to 735,709 bp, with an average of medians of 278,999.2 bp and an average median coverage of 85.2× (Table 1).

**TABLE 1:** Summary of the draft genome sequences for *Pseudomonas aeruginosa* environmental strains

Isolate	Origin	Location	No. of contigs	Genome size (bp)	G+C content (%)	*N*_50_ (bp)	Median read depth (×)	GenBank accession no.
Pae85	Soil	King City, Ontario, Canada	100	6,360,006	65.95	140,050	29	NKYD00000000
Pae100	Soil	King City, Ontario, Canada	74	6,436,962	66.42	196,319	26	NKYC00000000
Pae102	Soil	King City, Ontario, Canada	90	6,437,018	66.42	121,945	20	NKYB00000000
Pae110	Sediment	Toronto, Ontario, Canada	75	6,438,971	66.42	234,265	29	NKYA00000000
Pae111	Soil	Maysville, KY	71	6,385,725	66.47	206,050	22	NKXZ00000000
Pae112	Soil	Maysville, KY	68	6,444,483	66.39	231,479	40	NKXY00000000
Pae113	Sediment	Maysville, KY	65	6,393,508	66.47	245,535	45	NKXX00000000
Pae160	Water	South Africa	98	6,836,824	66.01	188,795	30	NKXW00000000
PaEB1	Water	Annandale, NY	65	6,336,070	66.50	735,709	337	NKXV00000000
PaEB6	Water	Annandale, NY	93	6,467,958	66.41	489,845	274	NKXU00000000

### Accession number(s).

The whole-genome shotgun projects have been deposited at DDBJ/ENA/GenBank (see Table 1). The versions described in this paper are NKYD01000000 (Pae85), NKYC01000000 (Pae100), NKYB01000000 (Pae102), NKYA01000000 (Pae110), NKXZ01000000 (Pae111), NKXY01000000 (Pae112), NKXX01000000 (Pae113), NKXW01000000 (Pae160), NKXV01000000 (PaEB1), and NKXU01000000 (PaEB6).
